# Always on My Mind? Recognition of Attractive Faces May Not Depend on Attention

**DOI:** 10.3389/fpsyg.2016.00053

**Published:** 2016-01-29

**Authors:** André Silva, António F. Macedo, Pedro B. Albuquerque, Joana Arantes

**Affiliations:** ^1^Human Cognition Laboratory, Department of Basic Psychology, School of Psychology, University of MinhoBraga, Portugal; ^2^Department of Physics, School of Sciences, University of MinhoBraga, Portugal

**Keywords:** attention, memory, recognition, attractiveness, eye tracking, eye-gaze, evolutionary psychology

## Abstract

Little research has examined what happens to attention and memory as a whole when humans see someone attractive. Hence, we investigated whether attractive stimuli gather more attention and are better remembered than unattractive stimuli. Participants took part in an attention task – in which matrices containing attractive and unattractive male naturalistic photographs were presented to 54 females, and measures of eye-gaze location and fixation duration using an eye-tracker were taken – followed by a recognition task. Eye-gaze was higher for the attractive stimuli compared to unattractive stimuli. Also, attractive photographs produced more hits and false recognitions than unattractive photographs which may indicate that regardless of attention allocation, attractive photographs produce more correct but also more false recognitions. We present an evolutionary explanation for this, as attending to more attractive faces but not always remembering them accurately and differentially compared with unseen attractive faces, may help females secure mates with higher reproductive value.

## Introduction

That are those who say that attractiveness is all in the eye of the beholder, but what is attractiveness anyway and why is it important? And also, what makes someone attractive? Has attractiveness any influence in us?

Research tells us that to maximize his or her reproductive success an individual must select a valuable mate (Pflüger et al., 2012), ensuring the maximization of the couple’s chances of successfully producing viable offspring, and raise and protect them when they are most vulnerable. Choosing a mate involves multiple processes, such as evaluating one’s own mate value relative to others, mate availability, congruency between individuals’ beliefs, among others (Buss, 2003), and entails indicators of mate value such as physical and facial attractiveness (e.g., Rhodes et al., 2005; Little et al., 2006; Prokop and Fedor, 2011). Indeed, both adults (Langlois et al., 2000) and infants (e.g., Rubenstein et al., 1999) seem to show a preference for attractive rather than unattractive faces. Attractiveness, also, provides information relevant for reproduction, including mates’ health (Boothroyd et al., 2013; Lee et al., 2013); mate quality (Pisanski and Feinberg, 2013; Doll et al., 2014); strength and dominance (e.g., Re et al., 2013); personality (e.g., Penton-Voak et al., 2006; Welling et al., 2009); intelligence (e.g., Kanazawa and Kovar, 2004; Denny, 2008); success (Lerner and Lerner, 1977); income (Frieze et al., 1991; Escasa et al., 2010) and emotional state (Adams and Kleck, 2003). Thus, being attractive seems to be advantageous to maximize reproductive success. Recent research on attractiveness and cognitive processes postulates that attractiveness increases our likelihood for differential reproduction by harnessing certain cognitive processes such as attention and memory (e.g., Maner et al., 2007; Anderson et al., 2010). As such, those who attend to attractive characteristics hold an advantage in successfully attaining a mate and producing offspring compared to others who are less prone to attend to attractiveness or to pair it with cues for health and other factors linked to better mate quality. In a similar way, individuals who better remember others with the advantageous characteristics will potentially give them more importance, and will adapt their behavior and expectations accordingly.

This became increasingly important throughout human evolution because although the majority of our early Pleistoscene ancestors were hunter gatherers and lived in small groups, it is also true that, namely during the Upper Pleistocene’s massive migrations, individuals from different communities met (Beyin, 2011). Indeed, recent research has shown that humans, Neanderthals and Denisovans have interbred to some extent (Abi-Rached et al., 2011; Currat and Excoffier, 2011; Condemi et al., 2013; Harris and Nielsen, 2015; Juric et al., 2015). Thus, different species of hominids were in contact with each other, despite belonging to separate communities. Moreover, there are even those who claim that concentrating on the Pleistocene is misleading, since adaptations and other evolutionary changes can arise in as little as 18 generations (or 450 years in the case of our species) (Buller, 2005). As such, being attentive to these characteristics may have already been an adaptive behavior, constituting a reproductive advantage, albeit small, later fixed as an adaptation to promote reproductive success. These pieces of evidence seem to show that humans, today, are the result of environmental and societal pressures across time, time that has not stopped in the Upper Pleistocene.

Considering the advantages brought by attractiveness, it seems that an underlying process arisen via evolutionary processes substantiates a common stereotype, which indicates that “What is beautiful is good” (e.g., Dion et al., 1972; Sigall and Ostrove, 1975; Brand et al., 2012). As Lorenzo et al. (2010) have put it “we [humans] do judge a book by its cover, but when it is beautiful, this also prompts us to read it more closely”. If attractive people are more likely to survive and reproduce, it can be speculated that attention should be biased toward them, as it would increase their salience, importance and processing priority, which, in turn, would reinforce their survival and reproducibility. One effect that seems to provide even more credibility to the importance of attractive faces is what Maner et al. (2007) called “attentional adhesion”, which can be defined as a hardwired capacity to process attractiveness to promote mate and rival awareness (see also, Maner et al., 2003; Hoss et al., 2005; Liu and Chen, 2012). Related to this, some researchers have claimed that attractiveness has a priority in terms of stimulus processing (Chen et al., 2012), even when the stimulus is stationary instead of moving (Anderson et al., 2010). Other studies (Duncan et al., 2007; Maner et al., 2007, 2012) also argue that the attentional processes depend upon the gender of the participant and whether they are interested in a short or long-term relationship, or have a restricted or unrestricted socio-sexual orientation.

Since these dynamics are observed in daily life, it is important to note that these results also hold true in ecological experiments. Lorenzo et al. (2010) had 73 undergraduate students (56 of whom female) complete a questionnaire assessing their personality traits and intelligence. The authors observed that physically attractive individuals were viewed more positively after three minutes of interaction. Additionally, attractive individuals were viewed with greater normative and distinctive accuracy and as having more positive and unique characteristics. Of interest, unattractiveness was not associated with decreases in accuracy. Therefore, it seems that unattractive individuals may represent the baseline for accuracy, rather than belonging to the negative pole. Overall, attractiveness seems to produce an increase - in number or in degree – of desirable characteristics, which can enhance relationship’s quality perceptions. Other authors (Valentine et al., 2014), drawing data from a speed dating study, developed a model in which greater facial width-to-height ratio lead to perceptions of higher dominance. This altered perception caused women to find men attractive, which resulted in a bigger interest in men for short-term relationships but not for long-term relationships. These results seem to provide further support to some evolutionary explanations, namely that human attention has been adaptively tuned to cues that help solve fitness-relevant problems, such as mating (Schaller et al., 2007). The only exceptions to this pattern were articles related to females suffering from eating disorders (Jansen et al., 2005; Horndasch et al., 2012; Greenberg et al., 2014) and to females who had higher than average body mass indices (Roefs et al., 2008): these participants were more attentive toward unattractive characteristics.

In the previous paragraphs we tried to make the case in favor of the idea that attention helps an individual focus on a potential mate, acknowledging the mate’s value and triggering the appropriate cognitive and behavioral responses, such as increasing its salience to promote mate awareness and to initiate action. An opposite-sex person can momentarily gather our attention, but after that moment a visual presence of the person ceases to be possible. Humans as well as other species (e.g., Dias and Ressler, 2014) have memory processes that may help retain part of fitness-relevant information. These memory processes increase the salience of the person, also increasing the likelihood of he/she being considered as a potential mate. If attractiveness captures our attention and is linked to several mate characteristics, it seems possible that we have evolved to retain attractive persons in our memory so that we can employ our resources toward pairing with that valuable person. Due to the dynamics of ancestral life, these processes may not have been as important as they are today, but considering the former paragraphs it seems they still hold some fitness relevance. In fact, there is some evidence that memory may possess a specific adaptation for mate choice (Allan et al., 2012) and that faces can be the most immediate stimuli for attractiveness appraisal (Little et al., 2011). Many studies have addressed the effect of attractive and unattractive stimuli on human memory. However, they have brought mixed results. Whereas some researchers reported more accurate memory for attractive stimuli (e.g., Cross et al., 1971; Allan et al., 2012; Kajimura et al., 2014), others found the opposite (Light et al., 1981; Sarno and Alley, 1997; Wiese et al., 2014), some did not obtain a difference in memory accuracy (Brigham, 1990; Wickham and Morris, 2003; Anderson et al., 2010) and others obtained a mixture of both (Deblieck and Zaidel, 2003). It also seems that, compared with males, females are better at remembering same-sex faces when faces of both sexes are presented (Rehnman and Herlitz, 2007; Wang, 2013). In addition they are also better at remembering opposite-sex faces compared to males when only opposite-sex faces are presented (Herlitz and Lovén, 2013), which indicates a female own-gender bias but not a male own-gender bias.

If attractiveness is, as it seems, such an important feature for evaluating potential mates, and if attention is somewhat related to memory (Heisz et al., 2013), it follows that paying more attention to opposite-sex persons may produce an effect on memory, leading to better remembering and higher recognition rates. So why did different authors obtain different and sometimes opposite results? Some authors claim that this may be due to not controlling certain facial characteristics, namely distinctiveness and prototypicality (Light et al., 1981; Bruce et al., 1994; Marzi and Viggiano, 2010; Wiese et al., 2014), motivation (e.g., Maner et al., 2012; Skelly and Decety, 2012; Kajimura et al., 2014), and familiarity (e.g., Shepherd et al., 1991; Monin, 2003; Corneille et al., 2005; Edmonds et al., 2012; Estudillo, 2012). Monin (2003) asked university students to rate 80 pictures on various dimensions, including attractiveness, familiarity, unfamiliarity and distinctiveness, and found that people rated the most attractive were also considered as the most likely of having been seen on campus, even after controlling for distinctiveness and despite all photographs being of people unknown to the participants. In the subsequent experiment, the author presented to 50 undergraduates 80 pictures in two sets of identical attractiveness. In the first part of the experiment, participants completed a simple task were they had to indicate the sex of the person depicted in all pictures of one of the sets. Next, Monin (2003) presented them with all 80 pictures and asked whether the pictures were old or new. His results show that the more attractive the faces were, the more likely they were classified as being “old” independent of them actually having being presented before. Of interest, the author claims that higher levels of attractiveness did not yield lower discriminability but instead led participants to rely on lower criteria to decide whether they have seen the faces before.

Not many studies have examined memory for attractive and unattractive faces taking into account attention (e.g., Maner et al., 2003; Anderson et al., 2010; Sakaki et al., 2012). Anderson et al. (2010) presented to 112 females four slides, each containing eight neutrally expressive faces in a counterbalanced combination of male, female, attractive, and average faces during which participants’ eye-movements were recorded. Next, participants completed a memory test consisting of all faces previously presented, plus an equal number of distractors, in which they had to respond using a six-point scale ranging from “Definitely did not see” to “Definitely did see”. The results showed that participants paid more attention to attractive faces compared to the average face, and that memory accuracy was higher for the attractive faces. However, these results only took into consideration memory accuracy, a measure of recognition sensitivity that controls for false recognitions, instead of detailing separately both hits and false recognitions (lower accuracy can mean high number of hits and of false recognitions or low number of hits and of false recognitions). In another report, Maner et al. (2003) included five studies, the first four focusing on attention and the last one on memory. Although the authors did analyze false recognitions in their last study, they divorced both cognitive processes since this study was independent from the other four and did not include an attention task. In yet another study on memory and attention for attractive stimuli, Sakaki et al. (2012) attempted to show that biological emotional stimuli (emotional stimuli relevant to survival and reproduction, such as naked bodies) automatically affect cognitive processes such as attention and memory, whereas socially emotional stimuli require additional processing to modulate them. Their results showed that compared to socially emotional stimuli, biologically emotional images gather attention more strongly and enhance memory even with limited cognitive resources. However, these authors did not compare attention and memory results for attractive and unattractive photographs and did not use an eye-tracker to certify that participants were, in fact, fixating the stimuli being presented.

From the review of the relevant literature we can conclude and ask the following: (1) There seems to be a consensus that attractiveness influences attentional processes; (2) It is unclear, however, whether attractiveness influences recognition memory and in what direction; (3) There is insufficient evidence regarding whether these differences in recognition are influenced by previous attentional processes.

Considering these questions, our main aim was to evaluate whether attractive faces gather more attention than unattractive faces and whether attractive faces are better remembered compared to unattractive faces. Another aim was to evaluate if attractive faces produce more false recognitions than unattractive faces, a result which could indicate a generalization effect, this is: attractive faces may share more features between themselves compared to the features shared by unattractive faces, causing not only already seen before photographs to be recognized but also never before seen ones.

Therefore, in the present investigation an experiment including an attention task using an eye-tracker and a recognition task was devised. We expect that such design will allow not only separate analyses of what happens to human attention and memory when seeing attractive and unattractive people, but will also enable a discussion of memory findings taking into account the results from the attention task.

Accordingly, our research hypotheses are the following: (1) Attractive stimuli will gather more attention and will be better remembered than unattractive stimuli; (2) Attractive photographs will yield higher recognition rates than unattractive photographs; (3) The higher recognition rates for attractive photographs in the memory task depend on previous higher fixation durations on those photographs. This approach to studying attention and memory for stimuli with different degrees of attractiveness enables a better understanding of what happens to human memory when participants are presented with attractive and unattractive alternatives.

## Materials and Methods

### Participants

A convenience sample of 53 Caucasian undergraduate female students from a university in northern Portugal aged between 18 and 35 years (*M*_Age_ = 20.59, *SD*_Age_ = 3.90) participated in this study in exchange for course credit. All participants had normal or corrected-to-normal vision and were heterosexuals. This study was carried out in accordance with the recommendations of the University of Minho’s Ethics Committee with written informed consent from all subjects. The research presented in this article was approved by the aforementioned Committee. All subjects gave written informed consent in accordance with the Declaration of Helsinki.

### Materials

We obtained the photographs used in this experiment from online sources (e.g., Google search, modeling websites), and chose them only if they were naturalistic sharp color photographs, with a clear view of the face of Caucasian males and with a resolution of at least 319 × 193 pixels. Half of the photographs (*n* = 60) maximized attractive features (e.g., facial symmetry, masculinity), whereas the other half maximized unattractive features (e.g., disproportionate nose, asymmetry). A total of 120 photographs was thus selected. We then normalized their backgrounds to the same gray color and resized them to 319x193 pixels. After selection and normalization of the photographs we presented them to an independent sample (*N* = 52; *M*_Age_ = 23.94; *DP*_Age_ = 5.21) that rated each for attractiveness on a 7-point scale (ranging from 1, *extremely unattractive*, to 7, *extremely attractive*). We then separated all photographs into two groups, attractive and unattractive, each containing 60 items. The average ratings for the attractive and unattractive male photos were 5.39 (*DP* = 0.79) and 1.41 (*DP* = 0.46), respectively, *t*(51) = 34.73, *p* < 0.001.

Afterward, we created five 4 × 4 matrices, each displaying 16 male photographs, half of them attractive and the other half unattractive (4 columns by 4 rows). These 16 photographs correspond to the 16 regions defined to analyze eye-tracking data. The distribution of the photographs per matrix and their relative positions within each matrix were randomized. These matrices matched the stimulus presentation monitor resolution of 1680 × 1050 pixels.

Eye movements were monitored and recorded using a binocular, remote eye-tracker running at 250 Hz (SMI RED250, SensoMotoric Instruments GmbH, Teltow, Germany). The eye-tracker was attached to a 22-inch monitor that was used to present the stimuli. A second computer connected to the eye-tracker was used to control it. Eye-movement recordings were synchronized with stimulus presentation. The program to control the experiment was programmed in Matlab using both SMI’s SDK and elements of Psychophysics toolbox (Brainard, 1997; Kleiner et al., 2007).

Additionally, we administered to our participants a brief socio-demographic questionnaire, which included questions about age, gender, and sexual orientation, whether they had normal, corrected-to-normal or uncorrected vision, and relationship status (single or dating). To those that were involved in a relationship, we also asked to specify the type of relationship they were in (short or long term) and their relationship satisfaction (unsatisfied, neutral, or satisfied). For those not in a relationship, we also asked to specify if were seeking a relationship (not seeking relationship or seeking relationship).

### Procedure

**FIGURE 1:**
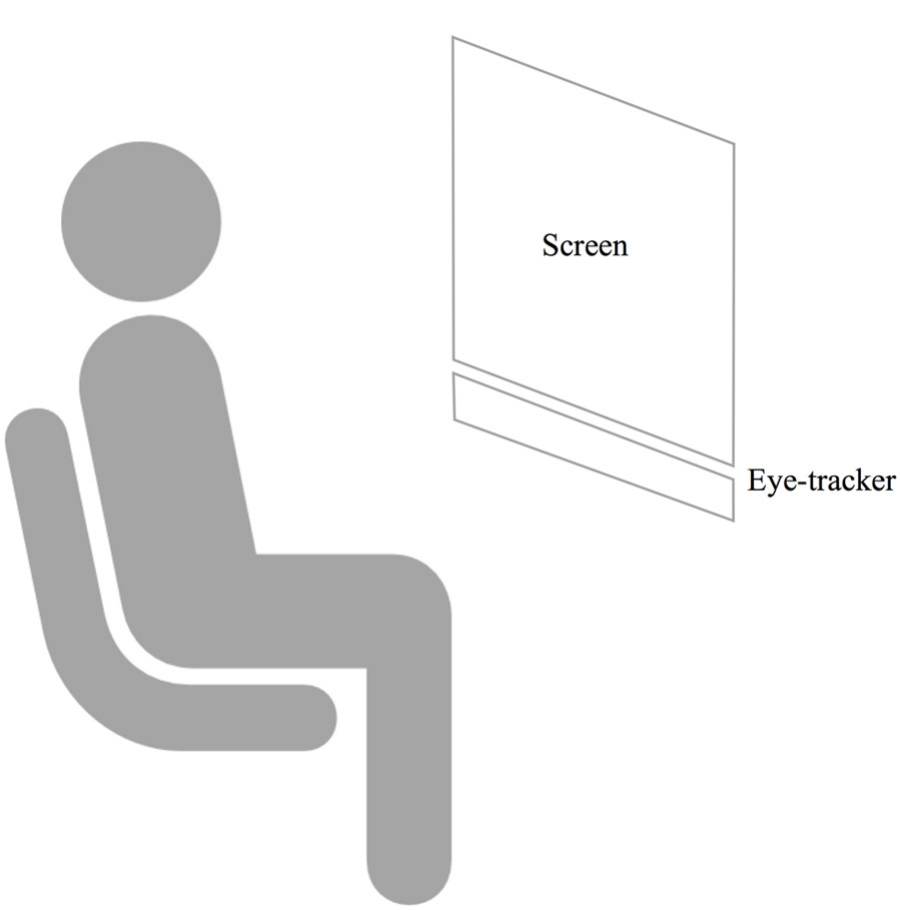
**Apparatus with participant, chair, screen, and eye-tracker.** Not seen are the table, computers, and peripherals.

Prior to the experimental phase, all participants filled the socio-demographic questionnaire. Participants were then seated in a fixed chair, positioned 70 cm away from the monitor and eye-tracker (see **Figure 1**). Before the presentation of the stimuli the eye-tracker was calibrated. A successful calibration corresponded to a mean spatial shift of 0.5° of visual angle between four points in the monitor and the position of the gaze when fixating those points.

**FIGURE 2:**
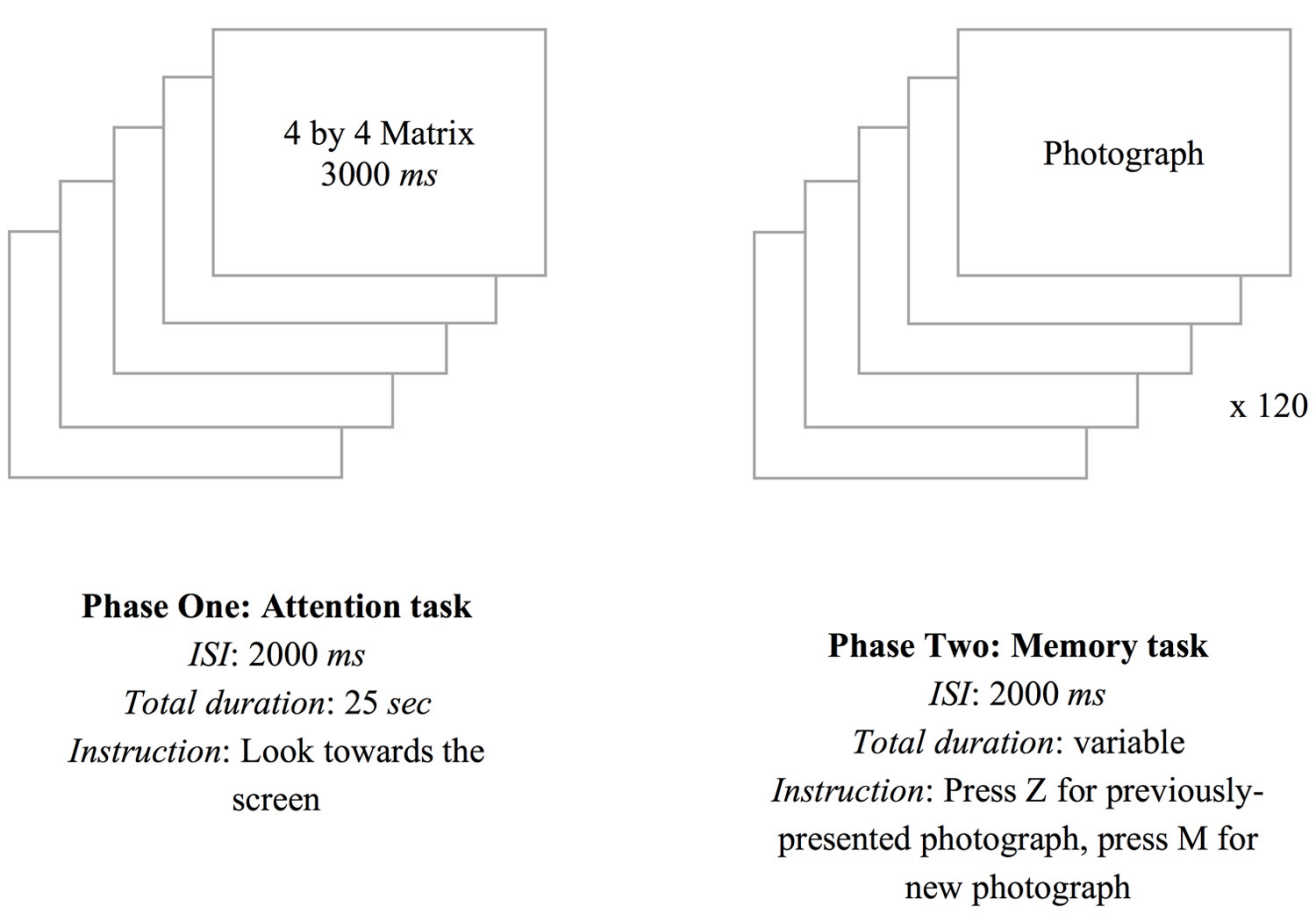
**Experimental procedure**.

The experiment proper consisted of two phases. In the first phase, we presented participants with all five matrices for three seconds each, and each matrix was followed by an inter-stimulus interval (ISI) of 2000 ms. During this phase we instructed the participants to look toward the screen. Unbeknown to the participants, this was done whilst their eye movements were monitored. In the second phase of the procedure, we presented all 120 photographs, one at a time at the center of the screen. Of these, 80 were equal to the ones previously presented in the matrices of phase one being the remaining 40 photographs 20 distractor attractive photographs and 20 distractor unattractive photographs. After being presented with each photograph, participants had to decide – by pressing the appropriate key on the keyboard – whether it was a new photograph or a previously presented photograph. There was no time limit to answer and, upon responding, a new photograph was presented (see **Figure 2** for a schematics). After all tasks were performed, participants were debriefed and thanked for their participation.

### Data Analysis

For the attention task, we analyzed eye movements oﬄine divided into two categories: saccades and fixations. A fixation can be defined as gaze on a particular location whereas a saccade can be defined as a fast movement between fixations. A saccade was defined when eye velocity was greater than 30° per second and/or acceleration was greater than 8500° per sec^2^. This threshold for filtering saccades from fixations has been used previously (Macedo et al., 2008), and is expected to detect only voluntary saccades including microssacades (involuntary saccades that interrupt fixation) as part of fixations (Martinez-Conde et al., 2006; Otero-Millan et al., 2014).

Regarding the memory task, we report our findings by analyzing responses on the memory task taking into account performance in the attention task. This means that all results presented below are for photographs that were shown during the attention task, which may or may not have been fixated upon. This analysis produces two kinds of responses, hits and false recognitions. A hit is made when participants fixate upon a photograph that was presented during the attention task and later correctly recognize it. A false recognition is made when participants do not fixate upon a photograph that was presented during the attention task but later incorrectly respond as having recognized it. Hits and false recognitions in this study are different from those commonly found in the literature, because in both cases the photographs are presented to the participants - normally a hit is a photograph that is presented and recognized, and a false alarm a photograph that wasn’t presented but was recognized. In this experiment we have an objective measure of attention due to the usage of an eye-tracker, allowing us to separate photographs according to whether or not they were fixated upon. Additionally, regardless of the participants’ fixating or not on the photographs presented within the matrices, we also analyzed all “I recognize” responses during this task, ignoring the eye-movements data collected during phase one. Participants’ response times (RT) during the memory task were also analyzed. A measure of sensitivity, such as *d prime* or other, was not included because the purpose of this study is to show whether participants tend to recognize attractive people more than unattractive people, regardless of that recognition being a truthful or false. This is something, we believe, was hinted at by Monin (2003).

For both tasks, we analyzed our results by group according to the socio-demographic variables: age, relationship status, relationship satisfaction, and desired relationship type.

## Results

Of our participants, 37 were in a relationship (69.8%), whereas 16 were single (30.2%). All those currently in a relationship considered it to be long-term (*n* = 37, 100%) and, overall, very satisfactory (*M*_Satisfaction_ = 4.41, *SD*_Satisfaction_ = 0.80). From those that were not currently in a relationship, five (31.3 %) were seeking a long-term relationship, two were seeking a short-term relationship (12.5 %), and nine were not seeking a relationship (56.3 %).

### Attention Task

#### Number of Fixations

**FIGURE 3:**
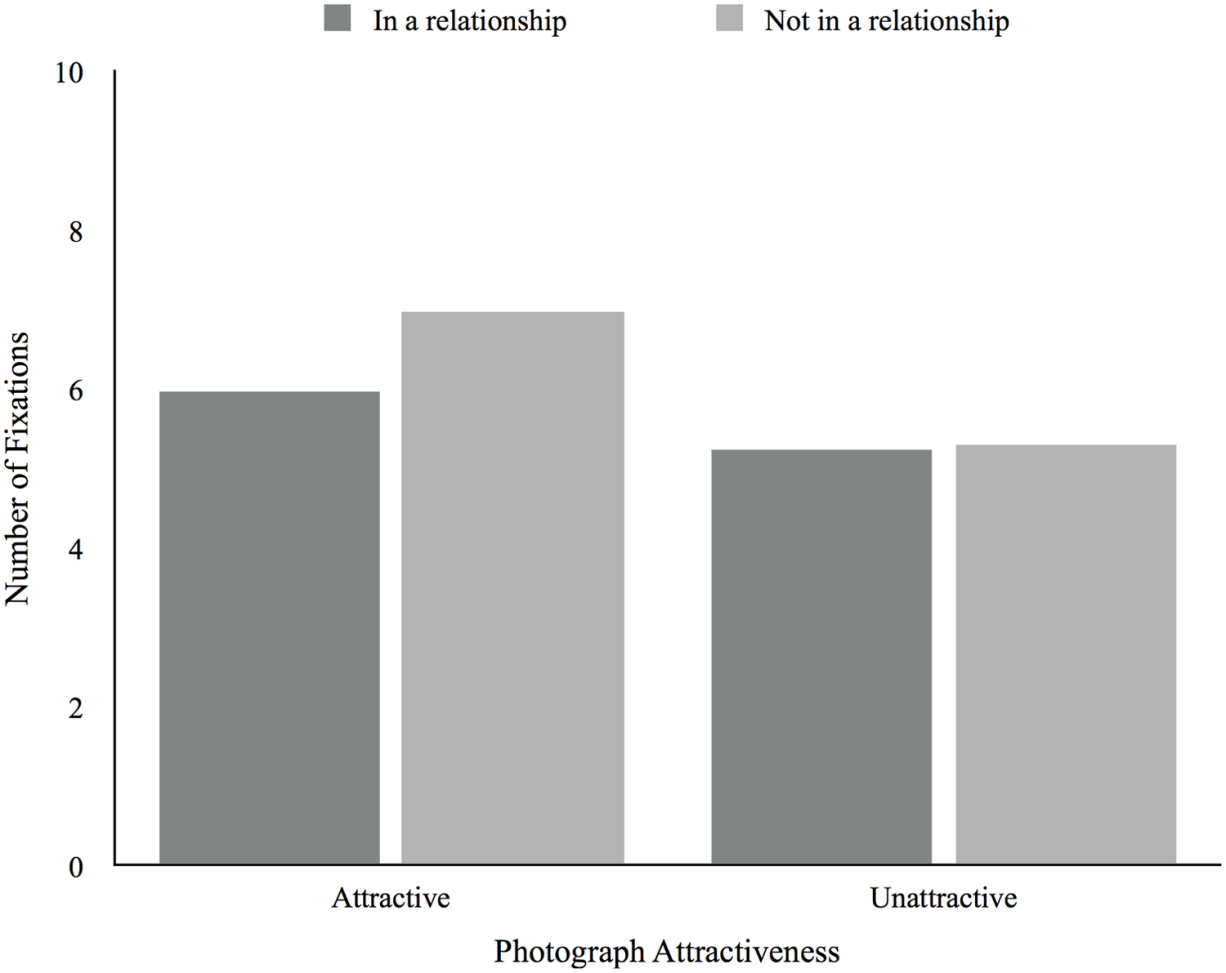
**Average number of fixations according to photograph attractiveness and relationship status**.

There was a main effect of attractiveness, *F*(1,51) = 13.46, *p* = 0.001, in which participants fixated more upon attractive photographs (*M* = 6.28, *SD* = 1.89) than unattractive photographs (*M* = 5.26, *SD* = 1.55). There was no main effect of relationship status, *F*(1,51) = 1.74, *p* = 0.19, and no attractiveness × relationship status interaction effect, *F*(1,51) = 2.15, *p* = 0.15 (see **Figure 3**). To better understand potential effects among each of these groups (those in a relationship and those not in a relationship), we analyzed relationship satisfaction for participants in a relationship and desired relationship type for those not in a relationship. There were no significant main effects of relationship satisfaction, *F*(3,33) = 0.53, *p* = 0.67, and desired relationship type, *F*(2,13) = 0.28, *p* = 0.76, and no significant attractiveness X relationship satisfaction, *F*(3,33) = 0.48, *p* = 0.70, and attractiveness × desired relationship type, *F*(2,13) = 0.59, *p* = 0.57, interaction effects. We found no correlations between age and number of fixations.

#### Fixation Duration (in Milliseconds)

**FIGURE 4:**
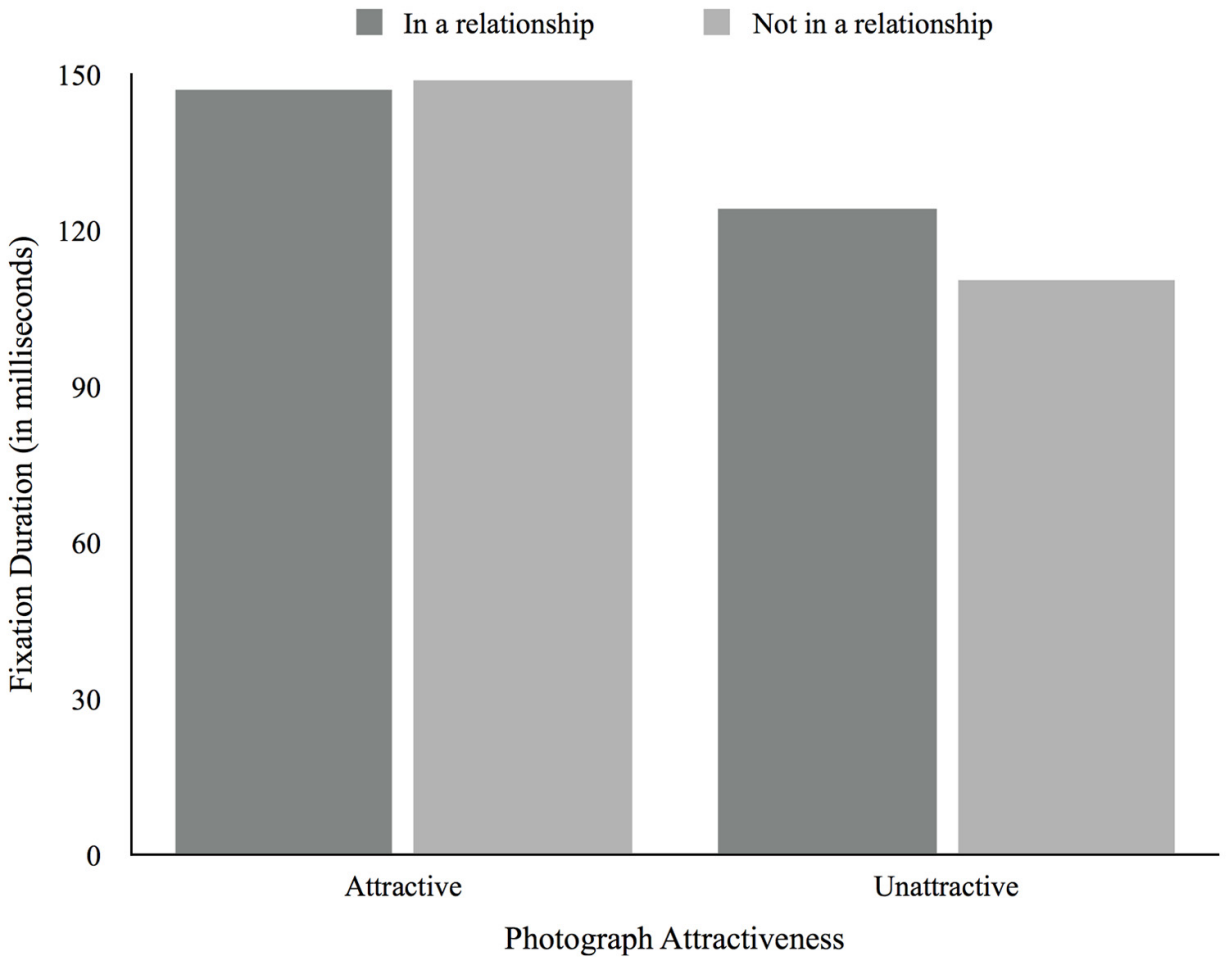
**Average fixation duration according to photograph attractiveness and relationship status**.

There was a main effect of attractiveness, *F*(1,51) = 13.72, *p* = 0.001, in which participants, regardless of relationship status, fixated attractive photographs for longer (*M* = 147.43, *SD* = 40.98) compared to unattractive photographs (*M* = 120.05, *SD* = 30.44). There was no main effect of relationship status, *F*(1,51) = 0.71, *p* = 0.41, and no attractiveness X relationship status interaction effect, *F*(1,51) = 0.90, *p* = 0.35 (see **Figure 4**). There were no significant main effects of relationship satisfaction, *F*(2,34) = 0.38, *p* = 0.69, and desired relationship type, *F*(1,14) = 1.11, *p* = 0.31, and no significant attractiveness × relationship satisfaction, *F*(2,34) = 0.01, *p* = 0.99, and attractiveness × desired relationship type, *F*(1,14) = 0.42, *p* = 0.53, interaction effects. We found no correlations between age and fixation duration.

### Memory Task

#### Number of Hits and False Recognitions

**FIGURE 5:**
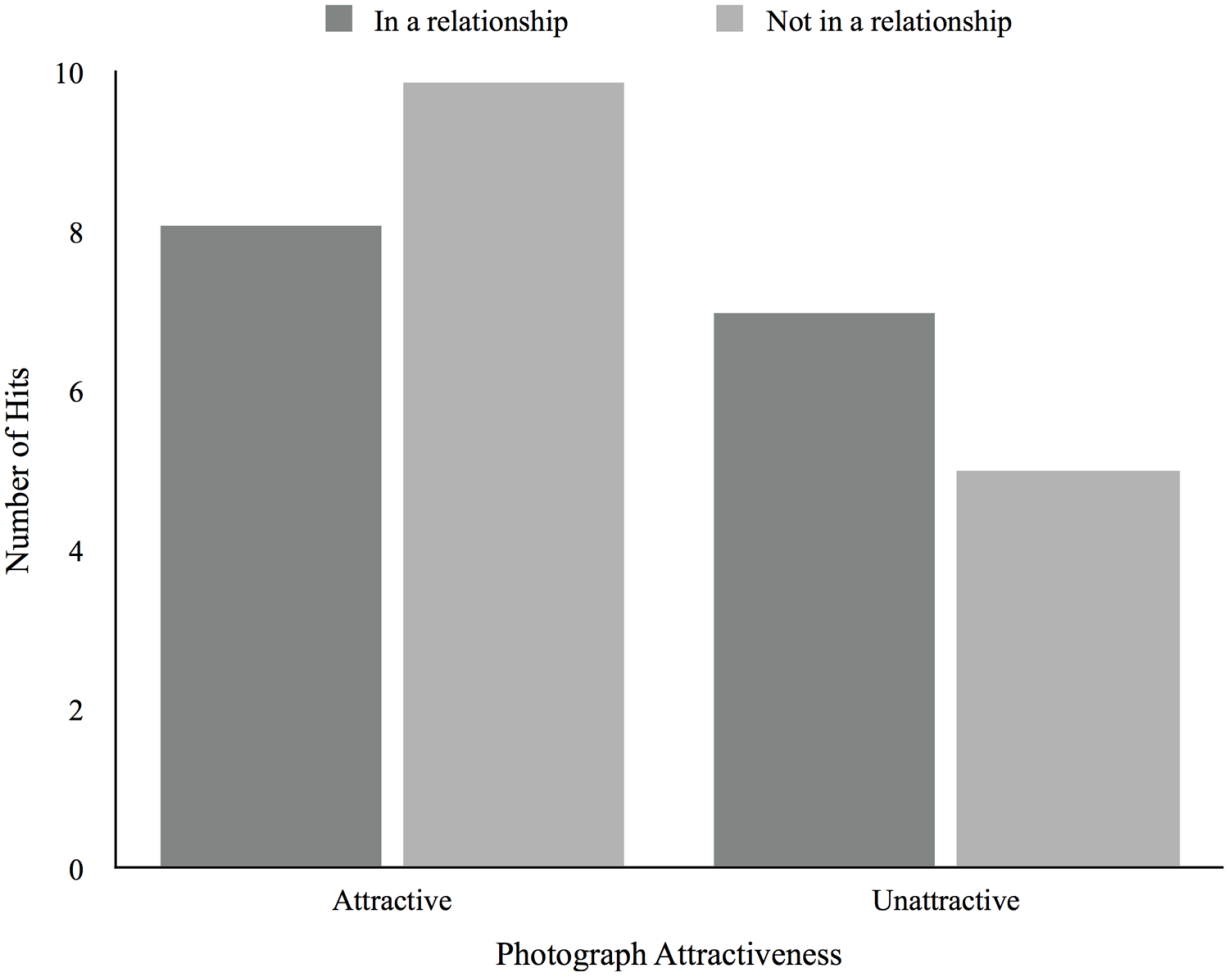
**Average number of hits according to photograph attractiveness and relationship status**.

**FIGURE 6:**
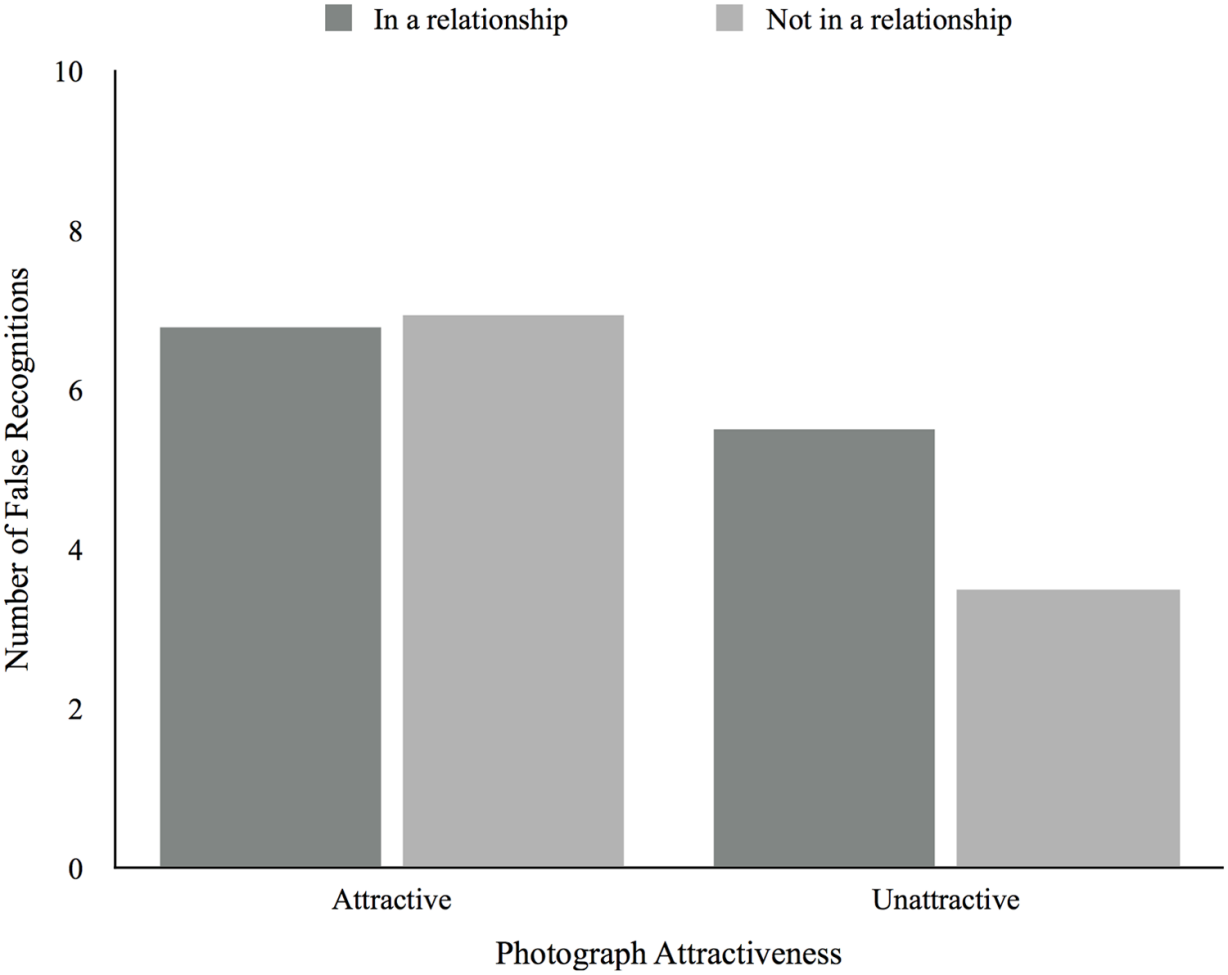
**Average number of false recognitions according to photograph attractiveness and relationship status**.

For the number of hits there was a main effect of attractiveness, *F*(1,50) = 10.28, *p* < 0.01, in which participants produced more hits for attractive photographs (*M* = 8.62, *SD* = 4.07) compared to unattractive photographs (*M* = 6.37, *SD* = 4.11). The same is true for false recognitions, *F*(1,50) = 10.91, *p* < 0.01, in which attractive photographs produced more false recognitions (*M* = 6.83, *SD* = 4.77) than did unattractive photographs (*M* = 4.88, *SD* = 4.01). There were no main effects of relationship status for both hits, *F*(1,50) = 0.01, *p* = 0.922, and false recognitions, *F*(1,50) = 0.68, *p* = 0.41. As for the interaction between attractiveness and relationship status it was significant for hits, *F*(1, 50) = 4.16, *p* = .05, in which those not in a relationship produced more hits for attractive (*M* = 9.88, *SD* = 1.01) compared to unattractive photographs (*M* = 5.00, *SD* = 1.01), being this difference in the same direction but greater than the one found for those in a relationship (*M_Attractive_* = 8.06, *SD* = 0.67; *M_Unattractive_* = 6.97, *SD* = 0.68; **Figure 5**). The interaction effect was not significant for the number of false recognitions, *F*(1,50) = 2.29, *p* = 0.14 (see **Figure 6**). There were no main effects of relationship satisfaction for both hits, *F*(2,33) = 1.23, *p* = 0.31, and false recognitions, *F*(2,33) = 3.08, *p* = 0.06, and no interaction effects between attractiveness and relationship satisfaction for hits, *F*(2,33) = 0.18, *p* = 0.83, and false recognitions, *F*(2,33) = 2.89, *p* = 0.07. There were also no main effects of desired relationship type for both hits, *F*(1,14) = 0.42, *p* = 0.53, and false recognitions, *F*(1,14) = 0.15, *p* = 0.71, and no interaction effects between attractiveness and desired relationship type for hits, *F*(1,14) = 0.25, *p* = 0.62, and false recognitions, *F*(1,14) = 0.05, *p* = 0.83. We found no correlations between age and the number of hits and false recognitions.

After controlling for number of fixations, there was no main effect of attractiveness on the number of hits, *F*(1,50) = 3.85, *p* = 0.06, and of false alarms, *F*(1,50) = 1.38, *p* = 0.25.

#### Correlations Between Attention Allocation and Number of Hits and False Recognitions

There were significant correlations between attention allocation toward attractive photographs and number of hits, *r* = 0.36, *p* = 0.01, and between unattractive photographs and number of hits, *r* = 0.39, *p* < 0.01. There were also significant correlations between number of hits and number of false recognitions for both attractive, *r* = 0.43, *p* < 0.01, and unattractive photographs, *r* = 0.35, *p* = 0.01. In addition, there was a significant correlation between attention allocation toward attractive photographs and number of hits for unattractive photographs, *r* = –0.48, *p* < 0.001.

#### Response Times for Hits and False Recognitions (in Milliseconds)

**FIGURE 7:**
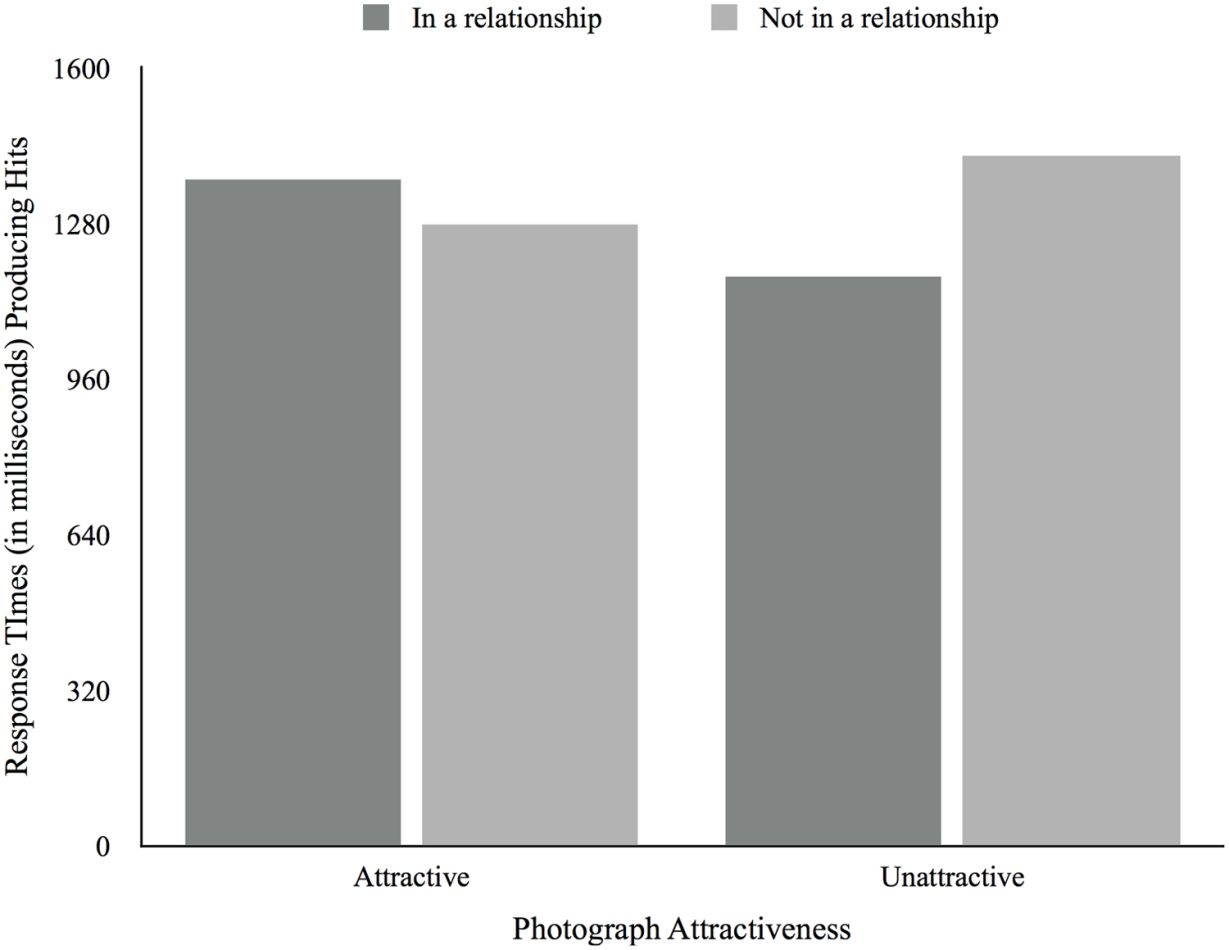
**Average response times producing hits according to photograph attractiveness and relationship status**.

**FIGURE 8:**
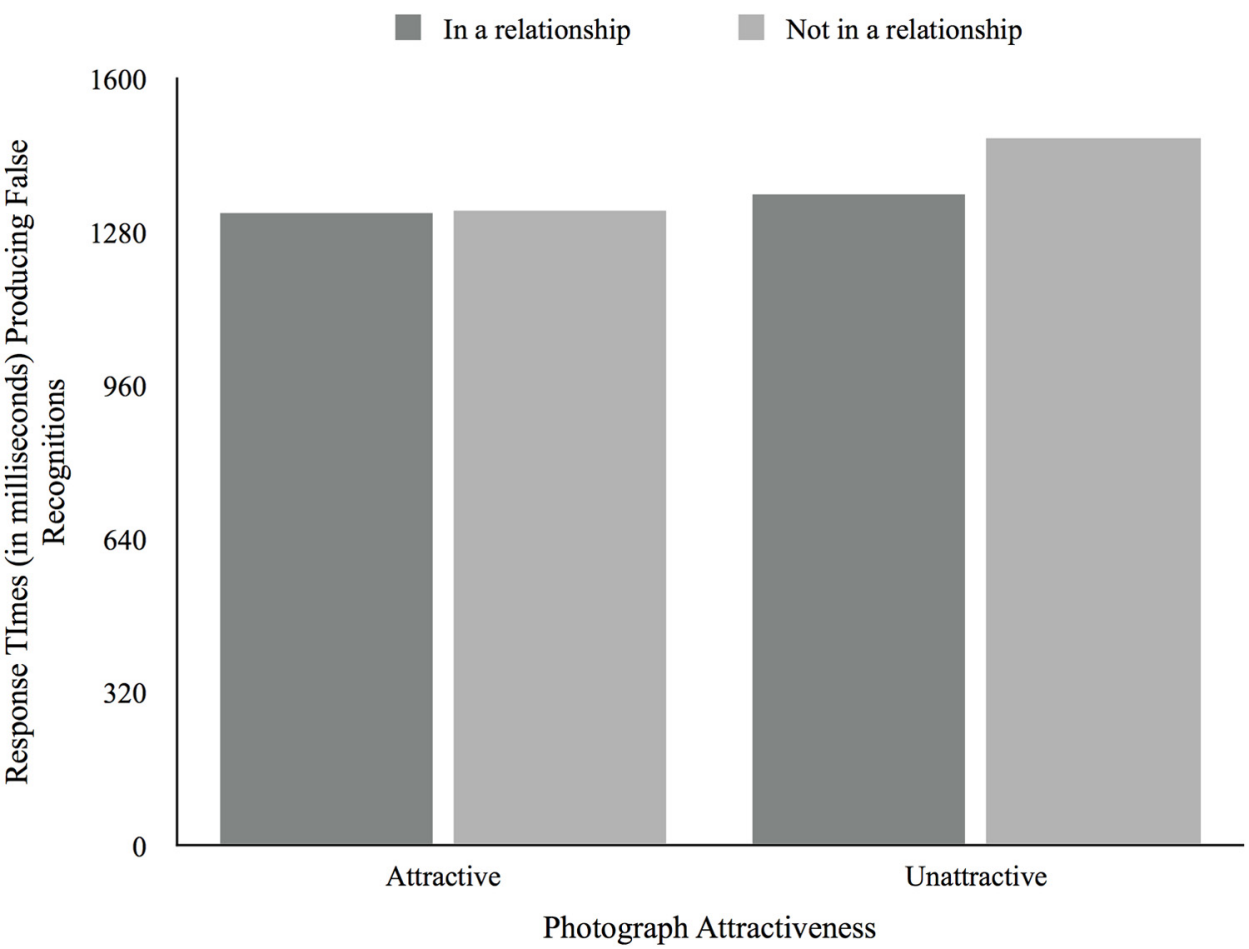
**Average response times producing false recognitions according to photograph attractiveness and relationship status**.

Next we analyzed the RTs. There was no main effect of attractiveness for RTs for both hits, *F*(1,45) = 0.16, *p* < 0.69, and false recognitions, *F*(1,45) = 1.16, *p* = 0.29. There was no significant main effect of relationship status for both hits, *F*(1,45) = 0.40, *p* = 0.53, and false recognitions, *F*(1,45) = 0.19, *p* = 0.66. However, there was a significant attractiveness × relationship status interaction effect for hits, *F*(1,45) = 4.59, *p* = 0.04, but not for false recognitions, *F*(1,45) = 0.41, *p* = 0.53 (see **Figures 7** and **8**). Participants not in a relationship took longer producing hits for unattractive photographs (*M* = 1417.36, *SD* = 95.06) compared to attractive photographs (*M* = 1279.47, *SD* = 148.47), while participants in a relationship took longer producing hits for attractive photographs (*M* = 1369.44, *SD* = 96.70) rather than unattractive photographs (*M* = 1168.26, *SD* = 61.91). There were no main effects of relationship satisfaction producing both hits, *F*(2,30) = 0.21, *p* = 0.81, and false recognitions, *F*(2,30) = 0.21, *p* = 0.81. There was a significant attractiveness × relationship satisfaction interaction effect for false recognitions, *F*(2,30) = 3.43, *p* = 0.05, but not for hits, *F*(2,30) = 0.33, *p* = 0.73. As such, while those unsatisfied or neutral with their relationships took longer producing false recognitions when presented with attractive photographs and less time when presented with unattractive photographs (*M_Unsatisfied_* = 2126.25, *SD_Unsatisfied_* = 503.15; *M_Neutral_* = 992.53, *SD_Neutral_* = 630.91), those participants satisfied with their relationships took less time producing false recognitions for attractive photographs (*M* = 1296.99, *SD* = 93.43) compared to unattractive photographs (*M* = 1390.53, *SD* = 117.16). Finally, there was no main effect of desired relationship type for both hits, *F*(1,12) = 1.21, *p* = 0.29, and false recognitions, *F*(1,12) = 0.00, *p* = 0.96. As for relationship satisfaction, there was a significant attractiveness X desired relationship type interaction effect for false recognitions, *F*(1,12) = 10.78, *p* = 0.01, but not for hits, *F*(1,12) = 0.20, *p* = 0.67. Thus, while those not seeking a relationship were faster producing false recognitions when presented with attractive photographs (*M* = 1129.33, *SD* = 176.38) and slower when presented with unattractive photographs (*M* = 1664.30, *SD* = 107.27), the opposite was true for those seeking a relationship (*M_Attractive_* = 1585.31, *SD_Attractive_* = 203.661; *M_Unattractive_* = 1227.47, *SD_Unttractive_* = 123.87). We found no correlations between age and the RT producing hits and false recognitions.

## Discussion

The main aim of this experiment was to analyze whether attractive stimuli receive more attention and are better remembered than unattractive stimuli, and whether this remembrance depends on previous attention allocation to attractive stimuli. We hypothesized that attractive stimuli would gather more attention and would be better remembered compared with unattractive stimuli, and that this better retrieval for attractive photographs would depend on greater fixation durations on the same photographs in the attentional task. According to our results, when participants are presented with attractive and unattractive photographs, attractive photographs are attended for longer and are fixated upon more compared with unattractive photographs, a result which is consistent with the literature (e.g., Hoss et al., 2005; Maner et al., 2007). As such, it seems that stimuli with attractive features have an attentional advantage, capturing, and retaining more attention than stimuli with unattractive features. This differential capture of attention may be due to attentional adhesion (Maner et al., 2007), a hardwired capacity to attend to attractive stimuli that may have evolved via natural and sexual selections to promote reproduction. Therefore by ensuring the attainment of a valuable mate (e.g., Pflüger et al., 2012), attention may be adaptively tuned to cues that help solve fitness-relevant situations, such as mate selection (e.g., Maner et al., 2003, 2007; Schaller et al., 2007).

The present results also show that hits are greater for attractive stimuli, which may suggest that memory is tuned to this kind of stimulus, as suggested by Allan and colleagues (2012). However, when considering false recognitions, participants also responded “I recognize” more when presented with attractive photographs compared with unattractive ones. This result suggests that when presented with both attractive and unattractive stimuli, participants tend to recognize attractive photographs more. Also, higher attention allocation produces higher number of hits for both attractive and unattractive photographs. Interestingly, the more one looks toward attractive photographs the less likely one is to recognize unattractive photographs. It thus appears that attractiveness, rather than mere attention allocation, may be responsible for these results.

A possible explanation for these results is that, as already noted for the advantage of attractive stimuli in attentional capturing and processing, it seems that attractive photographs have features that make participants produce more recognition responses. This does not mean that memory is necessarily better for these stimuli, but simply that attractiveness may make people think they recognize people with those features more than those without them. This effect makes evolutionary sense (Allan et al., 2012) because if attractive features indicate better mate quality, it may be useful for humans to not only pay more attention to attractive potential mates but also to better remember them, almost regardless of them having previously been seen. Specifically, in an ancestral world where meetings between non-kin individuals living farther apart existed in addition to those between kin members, to “remember” attractive potential mates would allow them to evaluate not only currently available mates but also those with whom they shared activities in the surrounding environment or those who, despite not having met, possess features that are advantageous.

Since not only hits but also false recognitions are higher for attractive photographs, we do not believe that later memory recognition further increases attention toward the same attractive photographs. Considering our evolutionary path, if recognition of previously seen attractive potential mates increased attention toward these – and only these –, false recognitions should be similar for attractive and unattractive photographs: we would be fine-tuned to recognize the attractive individuals we already met and gazed at for longer. This could make sense and lead to less energy expenditure since people would have a perfect tuning to an already seen ideal mate and therefore would not need to be always alert toward attractive features in others. However, if humans behaved as described, competition would be extreme and inevitable, as all individuals would be perfectly attracted to the same opposite-sex potential mates. To solve this problem, evolutionary processes may have shaped memory so that instead of being perfectly paired with attention, it is only partially so. In this way, humans pay more attention toward available attractive alternatives but do not recognize them much better than previously unseen attractive alternatives. We even venture arguing that this function of memory recognition exists to prevent attentional bias toward previously seen attractive potential mates; with this function no attractive alternative is excluded as potential for mating and all good options remain on the table. In other words, it seems that attractive potential mates may gather more attention than their unattractive counterparts, but regardless of attention given attractive potential mates may be considered as having “been there”, even when they were not. In addition, by functioning this way memory reduces further biases for attractive alternatives but the same may not be true for unattractive ones. Even when individuals pay no attention to unattractive alternatives - because that could distract them from valuable mates - they are better at rejecting new unattractive alternatives. It seems that the human brain thus produces a red flag: unattractive individuals are to be prevented from exhausting cognitive resources. This could be an explanation as for why our participants not only rejected less previously unseen attractive photographs compared with unattractive ones, but also took longer to reject previously unseen attractive photographs. Specifically, we hypothesize that part of the cognitive system “knows” that the photographs were not seen before. However, because the photographs are attractive, it may be useful to “remember” them, even though falsely. Considering previous research, it seems that we have to agree with those claiming that attractive stimuli have an advantage in enhancing memory recognition – such as Cross et al. (1971), Kajimura et al. (2014) and others (Marzi and Viggiano, 2010; Tsukiura and Cabeza, 2011; Zhang et al., 2011) – while also agreeing with those claiming that the accuracy of that recognition is lower for attractive stimuli (Light et al., 1981; Sarno and Alley, 1997; Wiese et al., 2014).

However, attention may bias our recognition of attractive faces – which may have the evolutionary explanation we proposed above – but we may recognize theses faces more merely for having seen them for longer. In fact, after controlling for number of fixations, there was no main effect of attractiveness on the number of hits and false recognitions. Being true, this means that more attention for attractive faces may well be due to evolutionary processes, but recognizing them more may be due to simply having seen them for longer. If this is true, however, both attractive and unattractive distractor faces – those not fixated upon during the attention phase – should, in principle, have the same pattern of recognition. This is not what we found, as attractive faces produce more false recognitions than unattractive faces. Memory, thus, may not depend entirely on attention. Isolating time spent gazing at the photographs would not allow participants to fixate upon the photographs that naturally gather more attention – in this case, attractive faces - possibly defeating the purpose of the experiment. However, we agree that it can be interesting to manipulate time spent gazing and discuss the results of such experiments. This is something the authors are considering for future research endeavors.

According to our results for the sociodemographic variables in the memory task, we concluded that participants that were not in a relationship produced more hits for attractive faces compared to those in a relationship, being that number higher than for unattractive faces, being also faster at producing hits for attractive faces. Thus, it seems that not being in a relationship may make participants attend more to attractive facial features so that when presented with attractive faces they tend to recognize them better and faster compared to those in a relationship. In addition, participants that were unsatisfied or neither satisfied nor unsatisfied with their relationships took longer producing false recognitions for attractive photographs whereas those satisfied took less time in producing false recognitions for attractive photographs. Participants that were not seeking a relationship were faster producing false recognitions for attractive photographs, whereas those seeking a relationship were faster for unattractive photographs.

## Limitations and Further Research

The present study has some limitations, one of which relates to the usage of photographs downloaded from various online sources and some features of the photographs that were not controlled for. Future studies could include fewer photographs retrieved from a single source, allowing for better control of the stimuli, or even employing computer-generated imagery, options that we are currently exploring. We also suggest that future research include different attention and memory paradigms, a more varied and cross-cultural sample as well as including variables such as socio-sexuality, sexual orientation, among others. Also, it is important to include both male and female participants viewing both same and opposite-sex photographs. This would ensure adequate generalization of the results, as it would take into consideration eventual gender differences in attention and memory for attractiveness, and possibly other important differences and interactions. We did ask our participants questions regarding relationship type and sexual orientation, but the typology and homogeneity of our sample – young heterosexual undergraduate females in long-term relationships - contributed to not allowing for the conduction of the tests that would allow us to ascertain if differences between these groups exist (e.g., only one gender, only one relationship type).

We also suggest that further studies include procedures that may help shed some light on the underlying processes or mechanisms by which previously seen attractive faces produce more recognition responses but also by which distractor attractive faces produce the same. Moreover, it would be interesting to keep fixation duration and number of fixations constant varying only attractiveness ratings, so that recognition would not also depend on that variable.

## Conclusion

By employing an attention task prior to a recognition task we were able to consider attentional processes and eye movements in false and truthful recognition. In sum, our results show that attention is biased toward attractive photographs rather than unattractive alternatives in static matrices. Moreover, our results show that memory does not work to promote recognition of previously seen attractive alternatives but rather to promote “remembering” attractive alternatives regardless of them having previously been seen. We hypothesize that this bias toward attractive faces may contribute to increase the salience of attractive individuals that have “better genes” for survival and reproduction, be them acquaintances or strangers from other social groups. This seems to be supported by the fact that participants that were not in a relationship fixated more upon attractive photographs compared to those that were in a relationship. In the same manner, this also seems to indicate that motivation and familiarity may play an important part in attractiveness appraisal and consequently on attention and on memory. Our findings extend a growing body of research and evidence implicating the adaptive function of cognitive processes such as attention and memory, suggesting that both work together albeit differently to differentially allocate resources to fitness-relevant stimuli, and thus promote reproductive success.

## Author Contributions

All authors listed, have made substantial, direct and intellectual contribution to the work, and approved it for publication.

## Conflict of Interest Statement

The authors declare that the research was conducted in the absence of any commercial or financial relationships that could be construed as a potential conflict of interest.

## References

[B1] Abi-Rached L. Jobin M. J. Kulkarni S. McWhinnie A. Dalva K. Gragert L. (2011). The shaping of modern human immune systems by multiregional admixture with archaic humans. *Science* 334 89–94. 10.1126/science.1209202 21868630 PMC3677943

[B2] Adams R. Kleck R. (2003). The integration of gaze direction and facial expression in the processing of facially communicated emotion. *Paper presented at the 4th Annual Meeting of the Society for Personality and Social Psychology*, Los Angeles, CA, February 6-8, 2003 10.1037/e633872013-852.

[B3] Allan K. Jones B. C. DeBruine L. M. Smith D. S. (2012). Evidence of adaptation for mate choice within women’s memory. *Evol. Hum. Behav.* 33 193–199. 10.1016/j.evolhumbehav.2011.09.002

[B4] Anderson U. S. Perea E. F. Vaughn Becker D. Ackerman J. M. Shapiro J. R. Neuberg S. L. (2010). I only have eyes for you: ovulation redirects attention (but not memory) to attractive men. *J. Exp. Soc. Psychol.* 46 804–808. 10.1016/j.jesp.2010.04.015 21874067 PMC3161129

[B5] Beyin A. (2011). Upper Pleistocene human dispersals out of Africa: a review of the current state of the debate. *Int. J. Evol. Biol.* 2011 1–17. 10.4061/2011/615094 PMC3119552 21716744

[B6] Boothroyd L. G. Scott I. Gray A. W. Coombes C. I. Pound N. (2013). Male facial masculinity as a cue to health outcomes. *Evol. Psychol.* 11 1044–1058. 10.1177/147470491301100508 24252513 PMC10434411

[B7] Brainard D. H. (1997). The psychophysics toolbox. *Spat. Vis.* 10 433–436. 10.1163/156856897X00357 9176952

[B8] Brand R. J. Bonatsos A. D’Orazio R. DeShong H. (2012). What is beautiful is good, even online: correlations between photo attractiveness and text attractiveness in men’s online dating profiles. *Comput. Hum. Behav.* 28 166–170. 10.1016/j.chb.2011.08.023

[B9] Brigham J. C. (1990). Target person distinctiveness and attractiveness as moderator variables in the confidence-accuracy relationship in eyewitness identifications. *Basic Appl. Soc. Psychol.* 11 101–115. 10.1207/s15324834basp1101_7

[B10] Bruce V. Burton M. A. Dench N. (1994). What’s distinctive about a distinctive face? *Q. J. Exp. Psychol. Sec. A* 47 119–141. 10.1080/14640749408401146 8177958

[B11] Buller D. J. (2005). *Adapting Minds: Evolutionary Psychology and the Persistent Quest for Human Nature*. Cambridge, MA: The MIT Press.

[B12] Buss D. M. (2003). *The Evolution of Desire: Strategies of Human Mating*. New York, NY: Basic Books.

[B13] Chen W. Liu C. H. Nakabayashi K. (2012). Beauty hinders attention switch in change detection: the role of facial attractiveness and distinctiveness. *PLoS ONE* 7:e32897 10.1371/journal.pone.0032897 PMC3290675 22393457

[B14] Condemi S. Mounier A. Giunti P. Lari M. Caramelli D. Longo L. (2013). Possible interbreeding in late Italian Neanderthals? New data from the Mezzena jaw (Monti Lessini, Verona, Italy). *PLoS ONE* 8:e59781 10.1371/journal.pone.0059781 PMC3609795 23544098

[B15] Corneille O. Monin B. Pleyers G. (2005). Is positivity a cue or a response option? Warm glow vs evaluative matching in the familiarity for attractive and not-so-attractive faces. *J. Exp. Soc. Psychol.* 41 431–437. 10.1016/j.jesp.2004.08.004

[B16] Cross J. F. Cross J. Daly J. (1971). Sex, race, age, and beauty as factors in recognition of faces. *Percept. Psychophys.* 10 393–396. 10.3758/BF03210319

[B17] Currat M. Excoffier L. (2011). Strong reproductive isolation between humans and Neanderthals inferred from observed patterns of introgression. *Proc. Natl. Acad. Sci. U.S.A.* 108 15129–15134. 10.1073/pnas.1107450108 21911389 PMC3174651

[B18] Deblieck C. Zaidel D. W. (2003). Hemifield memory for attractiveness. *Int. J. Neurosci.* 113 931–941. 10.1080/00207450390220358 12881186

[B19] Denny K. (2008). Beauty and intelligence may – or may not – be related. *Intelligence* 36 616–618. 10.1016/j.intell.2008.01.003

[B20] Dias B. G. Ressler K. J. (2014). Parental olfactory experience influences behavior and neural structure in subsequent generations. *Nat. Neurosci.* 17 89–96. 10.1038/nn.3594 24292232 PMC3923835

[B21] Dion K. Berscheid E. Walster E. (1972). What is beautiful is good. *J. Pers. Soc. Psychol.* 24 285–290. 10.1037/h0033731 4655540

[B22] Doll L. M. Hill A. K. Rotella M. A. Cárdenas R. A. Welling L. L. M. Wheatley J. R. (2014). How well do men’s faces and voices index mate quality and dominance? *Hum. Nat.* 25 200–212. 10.1007/s12110-014-9194-3 24578029

[B23] Duncan L. A. Park J. H. Faulkner J. Schaller M. Neuberg S. L. Kenrick D. T. (2007). Adaptive allocation of attention: effects of sex and sociosexuality on visual attention to attractive opposite-sex faces. *Evol. Hum. Behav.* 28 359–364. 10.1016/j.evolhumbehav.2007.05.001 17948071 PMC2034358

[B24] Edmonds E. C. Glisky E. L. Bartlett J. C. Rapcsak S. Z. (2012). Cognitive mechanisms of false facial recognition in older adults. *Psychol. Aging* 27 54–60. 10.1037/a0024582 21787088

[B25] Escasa M. Gray P. B. Patton J. Q. (2010). Male traits associated with attractiveness in Conambo, Ecuador. *Evol. Hum. Behav.* 31 193–200. 10.1016/j.evolhumbehav.2009.09.008

[B26] Estudillo A. J. (2012). Facial memory: the role of the pre-existing knowledge in face processing and recognition. *EJOP* 8 231–244. 10.5964/ejop.v8i2.455

[B27] Frieze I. H. Olson J. E. Russell J. (1991). Attractiveness and income for men and women in management. *J. Appl. Soc. Pyschol.* 21 1039–1057. 10.1111/j.1559-1816.1991.tb00458.x

[B28] Greenberg J. L. Reuman L. Hartmann A. S. Kasarskis I. Wilhelm S. (2014). Visual hot spots: an eye tracking study of attention bias in body dysmorphic disorder. *J. Psychiatr. Res.* 57 125–132. 10.1016/j.jpsychires.2014.06.015 25005739

[B29] Harris K. Nielsen R. (2015). The genetic cost of Neanderthal introgression. *bioRxiv* 1–17. 10.1101/030387 PMC4896200 27038113

[B30] Heisz J. J. Pottruff M. M. Shore D. I. (2013). Females scan more than males: a potential mechanism for sex differences in recognition memory. *Psychol. Sci.* 24 1157–1163. 10.1177/0956797612468281 23696202

[B31] Herlitz A. Lovén J. (2013). Sex differences and the own-gender bias in face recognition: a meta-analytic review. *Vis. Cogn.* 21 1306–1336. 10.1080/13506285.2013.823140

[B32] Horndasch S. Kratz O. Holczinger A. Heinrich H. Hönig F. Nöth E. (2012). “Looks do matter”—visual attentional biases in adolescent girls with eating disorders viewing body images. *Psychiatry Res.* 198 321–323. 10.1016/j.psychres.2011.12.029 22417927

[B33] Hoss R. A. Ramsey J. L. Griffin A. M. Langlois J. H. (2005). The role of facial attractiveness and facial masculinity/femininity in sex classification of faces. *Perception* 34 1459–1474. 10.1068/p5154 16457167 PMC1368665

[B34] Jansen A. Nederkoorn C. Mulkens S. (2005). Selective visual attention for ugly and beautiful body parts in eating disorders. *Behav. Res. Ther.* 43 183–196. 10.1016/j.brat.2004.01.003 15629749

[B35] Juric I. Aeschbacher S. Coop G. View M. (2015). The strength of selection against Neanderthal introgression. *bioRxiv* 1–17. 10.1101/030148 PMC5100956 27824859

[B36] Kajimura S. Himichi T. Nomura M. (2014). Beautiful faces enhance verbal working memory performance: an NIRS study. *Psychologia* 57 49–57. 10.2117/psysoc.2014.49

[B37] Kanazawa S. Kovar J. L. (2004). Why beautiful people are more intelligent. *Intelligence* 32 227–243. 10.1016/j.intell.2004.03.003

[B38] Kleiner M. Brainard D. Pelli D. (2007). What’s new in Psychtoolbox-3? *Perception* 36 ECVP Abstract Supplement.

[B39] Langlois J. H. Kalakanis L. Rubenstein A. J. Larson A. Hallam M. Smoot M. (2000). Maxims or myths of beauty? A meta-analytic and theoretical review. *Psychol. Bull.* 126 390–423. 10.1037/0033-2909.126.3.390 10825783

[B40] Lee A. J. Dubbs S. L. Kelly A. J. Hippel von W. Brooks R. C. Zietsch B. P. (2013). Human facial attributes, but not perceived intelligence, are used as cues of health and resource provision potential. *Behav. Ecol.* 24 779–787. 10.1093/beheco/ars199

[B41] Lerner R. M. Lerner J. V. (1977). Effects of age, sex, and physical attractiveness on child-peer relations, academic performance, and elementary school adjustment. *Dev. Psychol.* 13 585–590. 10.1037/0012-1649.13.6.585

[B42] Light L. L. Hollander S. Kayra-Stuart F. (1981). Why attractive people are harder to remember. *Pers. Soc. Psychol. Bull.* 7 269–276. 10.1177/014616728172014

[B43] Little A. C. Burt D. M. Perrett D. I. (2006). Assortative mating for perceived facial personality traits. *Pers. Individ. Dif.* 40 973–984. 10.1016/j.paid.2005.09.016

[B44] Little A. C. Jones B. C. DeBruine L. M. (2011). Facial attractiveness: evolutionary based research. *Philos. Trans. R. Soc. B Biol. Sci.* 366 1638–1659. 10.1098/rstb.2010.0404 PMC3130383 21536551

[B45] Liu C. H. Chen W. (2012). Beauty is better pursued: effects of attractiveness in multiple-face tracking. *Q. J. Exp. Psychol.* 65 553–564. 10.1080/17470218.2011.624186 22117090

[B46] Lorenzo G. L. Biesanz J. C. Human L. J. (2010). What is beautiful is good and more accurately understood: physical attractiveness and accuracy in first impressions of personality. *Psychol. Sci.* 21 1777–1782. 10.1177/0956797610388048 21051521

[B47] Macedo A. F. Crossland M. D. Rubin G. S. (2008). The effect of retinal image slip on peripheral visual acuity. *J. Vis.* 8 16.1–16.11. 10.1167/8.14.16 19146317

[B48] Maner J. K. Gailliot M. T. Rouby D. A. Miller S. L. (2007). Can’t take my eyes off you: attentional adhesion to mates and rivals. *J. Pers. Soc. Psychol.* 93 389–401. 10.1037/0022-3514.93.3.389 17723055

[B49] Maner J. K. Kenrick D. T. Becker D. V. Delton A. W. Hofer B. Wilbur C. J. (2003). Sexually selective cognition: beauty captures the mind of the beholder. *J. Pers. Soc. Psychol.* 85 1107–1120. 10.1037/0022-3514.85.6.1107 14674817

[B50] Maner J. K. Miller S. L. Moss J. H. Leo J. L. Plant E. A. (2012). Motivated social categorization: fundamental motives enhance people’s sensitivity to basic social categories. *J. Pers. Soc. Psychol.* 103 70–83. 10.1037/a0028172 22545747

[B51] Martinez-Conde S. Macknik S. L. Troncoso X. G. Dyar T. A. (2006). Microsaccades counteract visual fading during fixation. *Neuron* 49 297–305. 10.1016/j.neuron.2005.11.033 16423702

[B52] Marzi T. Viggiano M. P. (2010). When memory meets beauty: insights from event-related potentials. *Biol. Psychol.* 84 192–205. 10.1016/j.biopsycho.2010.01.013 20109520

[B53] Monin B. (2003). The warm glow heuristic: when liking leads to familiarity. *J. Pers. Soc. Psychol.* 85 1035–1048. 10.1037/0022-3514.85.6.1035 14674812

[B54] Otero-Millan J. Castro J. L. A. Macknik S. L. Martinez-Conde S. (2014). Unsupervised clustering method to detect microsaccades. *J. Vis.* 14 pii:18 10.1167/14.2.18 24569984

[B55] Penton-Voak I. S. Pound N. Little A. C. Perrett D. I. (2006). Personality judgments from natural and composite facial images: more evidence for a “kernel of truth” in social perception. *Soc. Cogn.* 24 607–640. 10.1521/soco.2006.24.5.607

[B56] Pflüger L. S. Oberzaucher E. Katina S. Holzleitner I. J. Grammer K. (2012). Cues to fertility: perceived attractiveness and facial shape predict reproductive success. *Evol. Hum. Behav.* 33 708–714. 10.1016/j.evolhumbehav.2012.05.005

[B57] Pisanski K. Feinberg D. R. (2013). Cross-cultural variation in mate preferences for averageness, symmetry, body size, and masculinity. *Cross-Cult. Res.* 47 162–197. 10.1177/1069397112471806

[B58] Prokop P. Fedor P. (2011). Physical attractiveness influences reproductive success of modern men. *J. Ethol.* 29 453–458. 10.1007/s10164-011-0274-0

[B59] Re D. E. DeBruine L. M. Jones B. C. Perrett D. I. (2013). Facial cues to perceived height influence leadership choices in simulated war and peace contexts. *Evolut. Psychol.* 11 89–103. 10.1177/147470491301100109 PMC10480859 23372088

[B60] Rehnman J. Herlitz A. (2007). Women remember more faces than men do. *Acta Psychol.* 124 344–355. 10.1016/j.actpsy.2006.04.004 16764811

[B61] Rhodes G. Simmons L. W. Peters M. (2005). Attractiveness and sexual behavior: does attractiveness enhance mating success? *Evol. Hum. Behav.* 26 186–201. 10.1016/j.evolhumbehav.2004.08.014

[B62] Roefs A. Jansen A. Moresi S. Willems P. van Grootel S. van der Borgh A. (2008). Looking good: BMI, attractiveness bias and visual attention. *Appetite* 51 552–555. 10.1016/j.appet.2008.04.008 18495295

[B63] Rubenstein A. J. Kalakanis L. Langlois J. H. (1999). Infant preferences for attractive faces: a cognitive explanation. *Dev. Psychol.* 35 848–855. 10.1037/0012-1649.35.3.848 10380874

[B64] Sakaki M. Niki K. Mather M. (2012). Beyond arousal and valence: the importance of the biological versus social relevance of emotional stimuli. *Cogn. Affect. Behav. Neurosci.* 12 115–139. 10.3758/s13415-011-0062-x 21964552 PMC3306241

[B65] Sarno J. A. Alley T. R. (1997). Attractiveness and the memorability of faces: only a matter of distinctiveness? *Am. J. Psychol.* 110 81–92. 10.2307/1423702

[B66] Schaller M. Park J. H. Kenrick D. T. (2007). “Human evolution and social cognition,” in *Oxford Handbook of Evolutionary Psychology*, eds Dunbar R. I. M. Barrett L. (Oxford: Oxford University Press), 491–504. 10.1093/oxfordhb/9780198568308.013.0033

[B67] Shepherd J. W. Gibling F. Ellis H. D. (1991). The effects of distinctiveness, presentation time and delay on face recognition. *Eur. J. Cogn. Psychol.* 3 137–145. 10.1080/09541449108406223

[B68] Sigall H. Ostrove N. (1975). Beautiful but dangerous: effects of offender attractiveness and nature of the crime on juridic judgment. *J. Pers. Soc. Psychol.* 31 410–414. 10.1037/h0076472

[B69] Skelly L. R. Decety J. (2012). Passive and motivated perception of emotional faces: qualitative and quantitative changes in the face processing network. *PLoS ONE* 7:e40371 10.1371/journal.pone.0040371 PMC3386961 22768287

[B70] Tsukiura T. Cabeza R. (2011). Remembering beauty: roles of orbitofrontal and hippocampal regions in successful memory encoding of attractive faces. *Neuroimage* 54 653–660. 10.1016/j.neuroimage.2010.07.046 20659568 PMC2962707

[B71] Valentine K. A. Li N. P. Penke L. Perrett D. I. (2014). Judging a man by the width of his face: the role of facial ratios and dominance in mate choice at speed-dating events. *Psychol. Sci.* 25 806–811. 10.1177/0956797613511823 24458269

[B72] Wang B. (2013). Gender difference in recognition memory for neutral and emotional faces. *Memory* 21 991–1003. 10.1080/09658211.2013.771273 23432017

[B73] Welling L. L. M. DeBruine L. M. Little A. C. Jones B. C. (2009). Extraversion predicts individual differences in women’s face preferences. *Pers. Individ. Dif.* 47 996–998. 10.1016/j.paid.2009.06.030

[B74] Wickham L. H. V. Morris P. E. (2003). Attractiveness, distinctiveness, and recognition of faces: attractive faces can be typical or distinctive but are not better recognized. *Am. J. Psychol.* 116 455–468. 10.2307/1423503 14503395

[B75] Wiese H. Altmann C. S. Schweinberger S. R. (2014). Effects of attractiveness on face memory separated from distinctiveness: evidence from event-related brain potentials. *Neuropsychologia* 56 26–36. 10.1016/j.neuropsychologia.2013.12.023 24406982

[B76] Zhang Y. Kong F. Chen H. Jackson T. Han L. Meng J. (2011). Identifying cognitive preferences for attractive female faces: an event-related potential experiment using a study-test paradigm. *J. Neurosci. Res.* 89 1887–1893. 10.1002/jnr.22724 21805493

